# Static moiré patterns in moving grids

**DOI:** 10.1038/s41598-020-70427-x

**Published:** 2020-09-02

**Authors:** Vladimir Saveljev, Jaisoon Kim, Jung-Young Son, Yongsuk Kim, Gwanghee Heo

**Affiliations:** 1 Public Safety Research Center, Konyang University, Nonsan, Chungcheongnam-do 32992 Republic of Korea; 2 Department of Physics, Myongji University, Yongin, Gyeonggi-do 17058 Republic of Korea; 3 Department of Medical IT Engineering, Konyang University, Daejeon, 35365 Republic of Korea; 4 Department of Civil and Environmental Engineering, Konyang University, Nonsan, Chungcheongnam-do 32992 Republic of Korea

**Keywords:** Optics and photonics, Applied optics, Displays

## Abstract

We describe an optical phenomenon of unmovable moiré patterns in sliding (moving) grids and gratings. The phenomenon was observed visually in the planar straight movement of the black-and-white gratings with a period of several mm. This is a velocity-independent effect confirmed analytically and in a computer simulation based on the spatial averaging. We found the static directions of the moiré patterns in the regular grids, but our technique can be also applied to other objects. The orientation and period of the static moiré patterns are not obvious, especially in the presence of the distance effect. The phenomenon can be practically used in security applications.

## Introduction

The moiré effect is an optical interaction (interference) between superposed layers with periodically modulated transmittance and close geometrical characteristics (period and orientation). The moiré patterns appear when two or more “repetitive structures (such as screens, grids or gratings) are superposed or viewed against each other”^1^. The mathematical representation of such interference is the point-by-point multiplication of the reflectance/transmittance functions of the superposed (overlapped) gratings.

In discussing the moiré effect, one cannot fail to mention fundamental papers and books by famous authors, such as I. Amidror, O. Kafri, K. Patorski, D. Post, O. Bryngdal, C.A. Sciammarella, P.S. Theocaris and other authors who laid a foundation of the moiré theory and applications. Their ground-breaking works^1–10^ made a great impact on the theoretical and experimental investigation of the moiré effect.

The moiré effect is well investigated in optics^1–3,6,11,12^ including the optical measurements^8,13^. Note that the involved gratings are not diffraction gratings, rather regular structures with a period of several mm or cm, which is much longer than the wavelength of the visible light. Therefore, the moiré effect can be described in terms of linear optics, i.e., by rays.

In visual displays (dynamic screens, still photographs, and printouts), the moiré effect may create an unwanted image overlaid with a useful image, because the moiré patterns may appear in some unintended areas of the screen; as a result, the image quality drops down. Therefore, in displays, the moiré effect is an undesirable visual effect^14,15^. This is especially important for autostereoscopic three-dimensional (3-D) displays^16^ because of their typical structure consisting of layers with close periods. This is a moiré effect in the macro world.

The moiré phenomenon can be observed not only under the visible light, but also in other rays, for instance, X-rays^17–19^, electron beams^20–23^, and infrared light^24,25^.

In the nano-world, the moiré effect has been, particularly, observed in nanoparticles under an electron microscope (EM). In nano-layers, the moiré patterns are often referred to as the moiré superstructure or superlattice^26,27^. The moiré effect in cylindrical nanoparticles, for instance, in single- and double-walled nanotubes (SWNT, DWNT) is also not unknown^28–30^.

The moiré effect can be used in the metrology to provide highly accurate measurements^31–34^. The investigation of the moiré effect for measurements can be found in^2–4,6–9^, besides, in the moiré interferometry^35,36^. Currently, this is a growing research/application area, some recent examples can be found in^37–39^.

The moiré effect can be efficiently used in many other application fields not connected to measurements directly, for example, in the alignment^40,41^, in the document protection^42–44^, in the image encryption^45,46^, as well as in 3D displays^47–49^ despite that typically in displays, it is a negative effect to be eliminated^14,15^.

In a linear (one-dimensional) structure, its physical characteristic (say, the optical density) is changed along a certain direction (a straight line) only. We will refer to one-dimensional planar structures consisting of repeated parallel lines as gratings. Structures consisting of repeated polygons will be referred to as grids. The grids are essentially two-dimensional structures; some of them can be represented as combinations of gratings, and some are equivalent to the regular tessellations^50,51^. When we will need to mention either grid or grating, we will use the word “object”. The three-dimensional moiré effect is considered in^2,3^ and, particularly, in common 3D shapes^52,53^. In the current paper, we only consider planar black-and-white, one- and two-dimensional objects of infinite size which consist of regularly repeated geometric elements.

**Figure 1:**
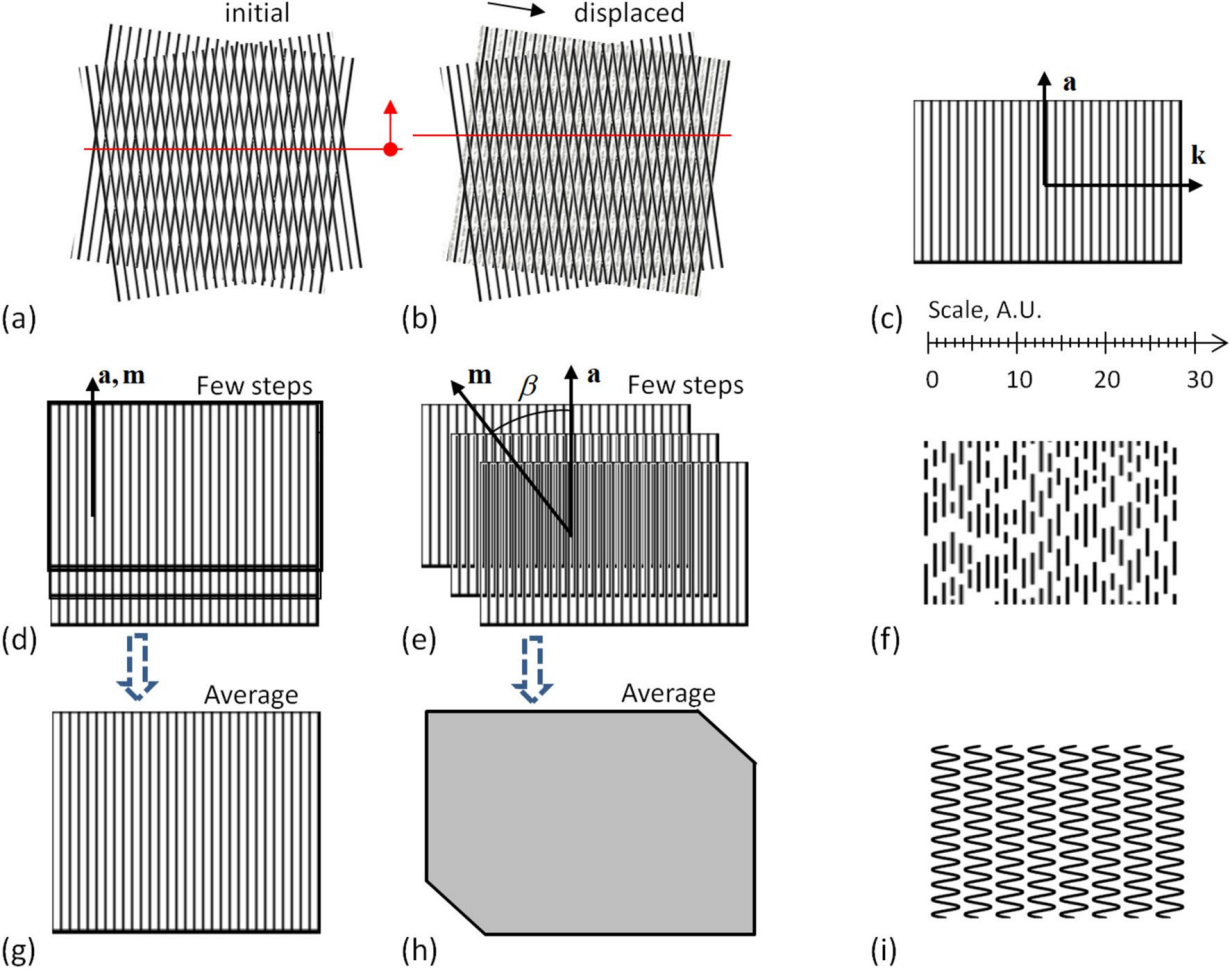
(**a**,**b)** Moiré patterns in line gratings, initial and displaced; the position of the patterns is shown by red lines. (**c**) Line grating, its wavevector **k** and the axis vector **a**. (**d**,**e**,**g**,**h**) Moved line grating with: (**d**) zero angle, (**e**) small angle. (**g**) Average visual picture of several steps with zero motion angle. (**h**) Average picture of grating sliding at an arbitrary angle (77°) with exposure distance (motion blur length) longer than period (fast move). (**f**,**i**) Gratings built of waved and dashed lines. The scale shown near section (**c**) is common for (**a**–**i**). (**a**,**b**) Moiré patterns in line gratings, initial and displaced; the position of the patterns is shown by red lines. (**c**) Line grating, its wavevector **k** and the axis vector **a**. (**d**,**e**,**g**,**h**) Moved line grating with: (**d**) zero angle, (**e**) small angle. (**g**) average visual picture of several steps with zero motion angle. (**h**) Average picture of grating sliding at an arbitrary angle (77°) with exposure distance (motion blur length) longer than period (fast move). (**f**,**i**) Gratings built of dashed and waved lines. The scale is common for (**a**–**i**).

The visual picture of the moiré patterns is determined by the relative position of the involved objects, see Fig. 1a. The displacement of an object by its period along the wavevector causes the displacement of the patterns by their period^1^; correspondingly, the displacement by one half of the period displaces the patterns by one half of their period, see Fig. 1b.

When an object moves, its visual appearance is the superposition of multiple elementary phases of movement during the exposure time of a camera; the moiré patterns also change. A dynamic picture depends, particularly, on the speed and direction of the motion.

With a few exceptions, the moiré effect is typically investigated in the static case. Some authors consider a dynamic or multiple-exposure effect^4,54,55^. The basic framework was established in^56^. The time-averaged moiré method in the application to vibrations is described in^57^. The time-averaged moiré patterns can be used in visual security^58^. The double-exposure of no-load and full-load interferometry patterns followed by the optical filtration is presented in^59^. The double-exposure technique is further developed in^60^, especially with the stroboscope^61^.

In a relatively slow movement, the path of the object during the time of the open camera shutter is less than the period of the geometric structure of the object; in this case, the visual picture becomes unstable and the patterns shift slowly, as illustrated in Supplementary Video S1 online. In the case of a relatively fast movement without stroboscopic illumination, the picture is blurred.

A sinusoidal grating of the infinite size is characterized by the amplitude *A*, the wavenumber *k* = 2π/*λ*, and the phase *φ*,

1 $$F = A\sin \left( {kx + \phi } \right)$$

A periodic grating of an arbitrary non-sinusoidal profile is characterized by the fundamental wavenumber and higher harmonics. In this paper, the word “fundamental” will be just omitted without losing essential details; we will simply say “wavevector” with no regards to the profile, sinusoidal or non-sinusoidal.

Define the axis vector **a** of the grating as the vector lying in the same plane with the grating and orthogonal to its wavevector **k**, see Fig. 1c; the vector **a** is directed along the lines of the line grating.

In the in-plane movement of the planar grating along the translation vector **m**, three mentioned vectors always lie in the same plane. The angle between the translation vector and the axis is *β*.

When the vectors **a** and **m** are collinear (see Fig. 1d), the displaced grating simply repeats itself and no visual change happens, as shown in Fig. 1g. When the angle *β* between **a** and **m** is small and the movement is slow, the picture of the grating starts to drift (see Supplementary Video S1 online). In the fast movement, the exposure time is longer than the time for the grating to move along several its periods as shown in Fig. 1e, and thus the image of the grating is averaged across the distance longer than the period; the result would look like a uniform gray field, see Fig. 1h. In the computer graphics, the directional spatial averaging can be modeled by the motion blur.

As soon as in this paper we consider averaging, a particular shape of the lines barely affects the visual picture. The lines only have to comply with a linear structure after averaging. Therefore in general, the lines may have a variety of forms: dashed, dotted, zigzag, waved, etc. For instance, when one of the gratings Fig. 1f,i is averaged along the vertical, the result remains similar to the line grating. At least, the profile of the averaged grating has a single maximum per period. We call such gratings approximate.

In grids (or in approximate line gratings shown in Fig. 1f,i), the averaging should be applied. The time-averaged moiré patterns are discussed in^57^ in connection with the periodic oscillations. Instead of the time averaging, we consider the spatial averaging along the direction of motion. We consider a relatively “fast” translation-only movement. From this point of view, it is a velocity independent effect at a high speed. The constancy of speed is not important.

At a few particular directions, the picture is not completely blurred to the uniform field; some traces of the original structure may remain, despite the motion of the gratings. We call such directions static directions. We found the condition for the moiré patterns to remain unmoved in the sliding gratings. In pure line gratings this is the exact effect, but in the approximate objects, the spatial averaging should be applied. We present the way how to identify the static directions in other periodic structures, e.g., in grids. We describe individual, collective, and coherent movement of objects.

Sometimes, such static patterns may look strange, counter-intuitive, and even paradoxical, especially when the movement of an object can be clearly seen.

Note that we did not apply a stroboscopic light or any other special (say, patterned) illumination. Neither had we applied image processing methods (such as a filtration) to improve characteristics of the resulting image such as the contrast. The static directions themselves could be practically useful in visual attractions and in security applications, e.g., in a counterfeit detection of valuable documents.

## Results

### Static directions in gratings

Generally, when a periodic grating of an infinite size moves along a certain angle *β* from its axis, such a movement can be treated in terms of the phase, i.e., as if the phase of the grating in Eq. (1) changes as

2 $$\phi = 2\pi \frac{v\left( t \right) \cdot t}{\lambda }\sin \beta$$

where *v* is the velocity, *t* is the time. In the case of a uniform linear movement, the velocity is constant and the phase is linearly proportional to the time,

3 $$\phi = 2\pi \frac{vt}{\lambda }\sin \beta$$

With an arbitrary angle *β*, the visual result is either drifted (lower speed and smaller *β*) or blurred (higher speed, larger *β*); the former case (drift) is illustrated in Supplementary Video S1 online. The only option for a grating to keep the constant phase with any speed is *β* = 0. Therefore, when a line grating moves exactly along its axis, such a movement does not change its appearance. The axis **a** is the only static direction of the grating (see Fig. 1c).

### Static directions in regular grids

In grids, however, other static directions may exist in addition to the axes of the involved elementary gratings. The static directions can be identified by analyzing averages at different directions, which can be obtained, for instance, by the Radon transform of a grid within a certain angular range; the result of the Radon transform is typically drawn as a 2D plot (the sinogram). Then, the static directions can be recognized as the angles (abscissas in the sinogram), where the vertical cross-section of the sinogram has a periodical structure. The period of the moiré patterns at that angle is equal to the period of the mentioned periodic structure.

**Figure 2:**
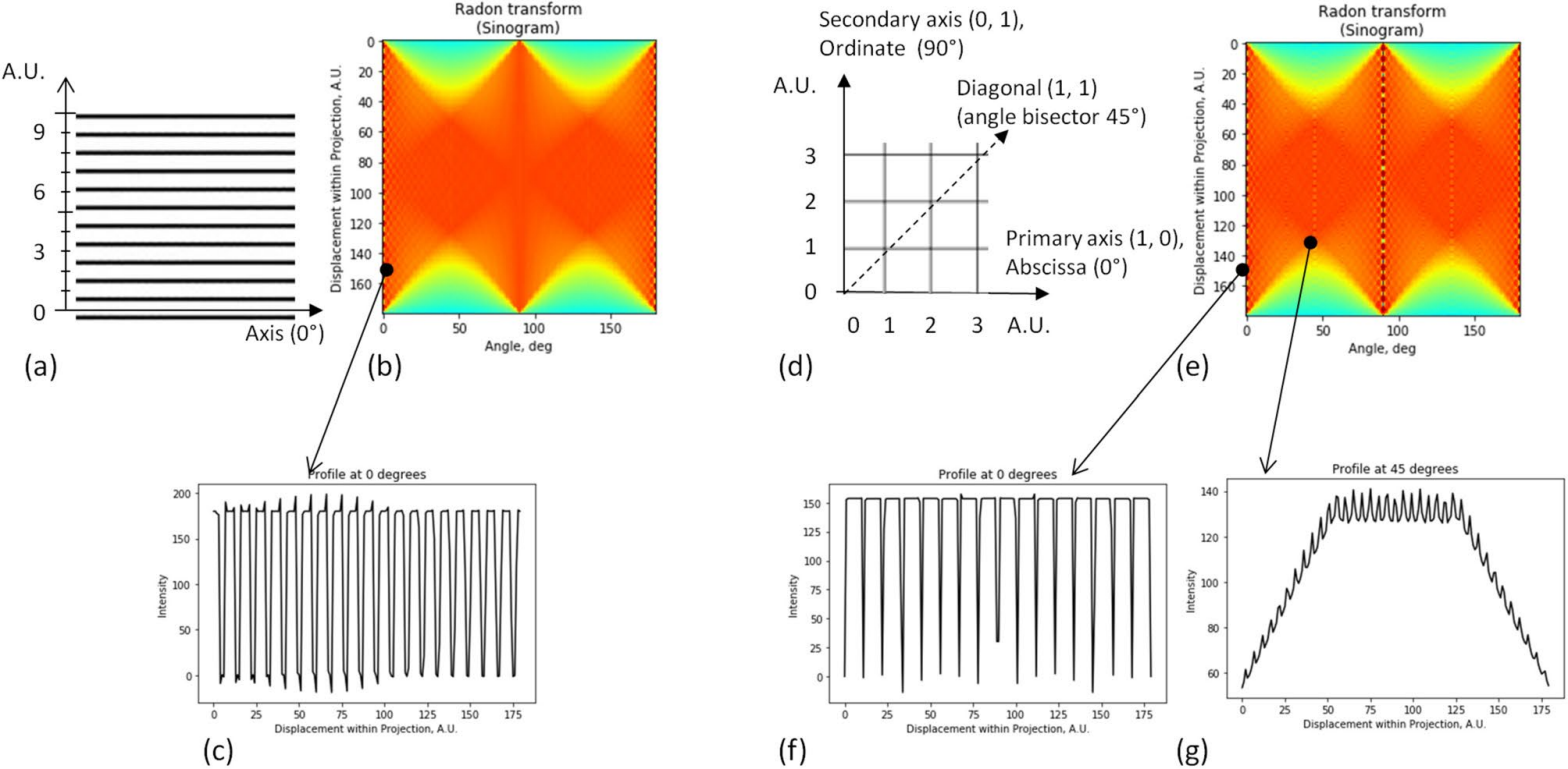
Radon transform of line grating and square grid. (**a**) Line grating. (**b**) Sinogram of Radon transform of line grating. Note the vertical periodic structures at the angles (abscissas) of 0 and 180°. (**c**) Profile of patterns along static direction (axis). (**d**) Square grid {4, 4} and significant directions in it. (**e**) Sinogram of square grid. (**f**,**g**) Profiles of patterns along two static directions: the highest contrast in (**f**), the lower contrast in (**g**). Due to the symmetry, the picture is repeated after 90°.

**Figure 3:**
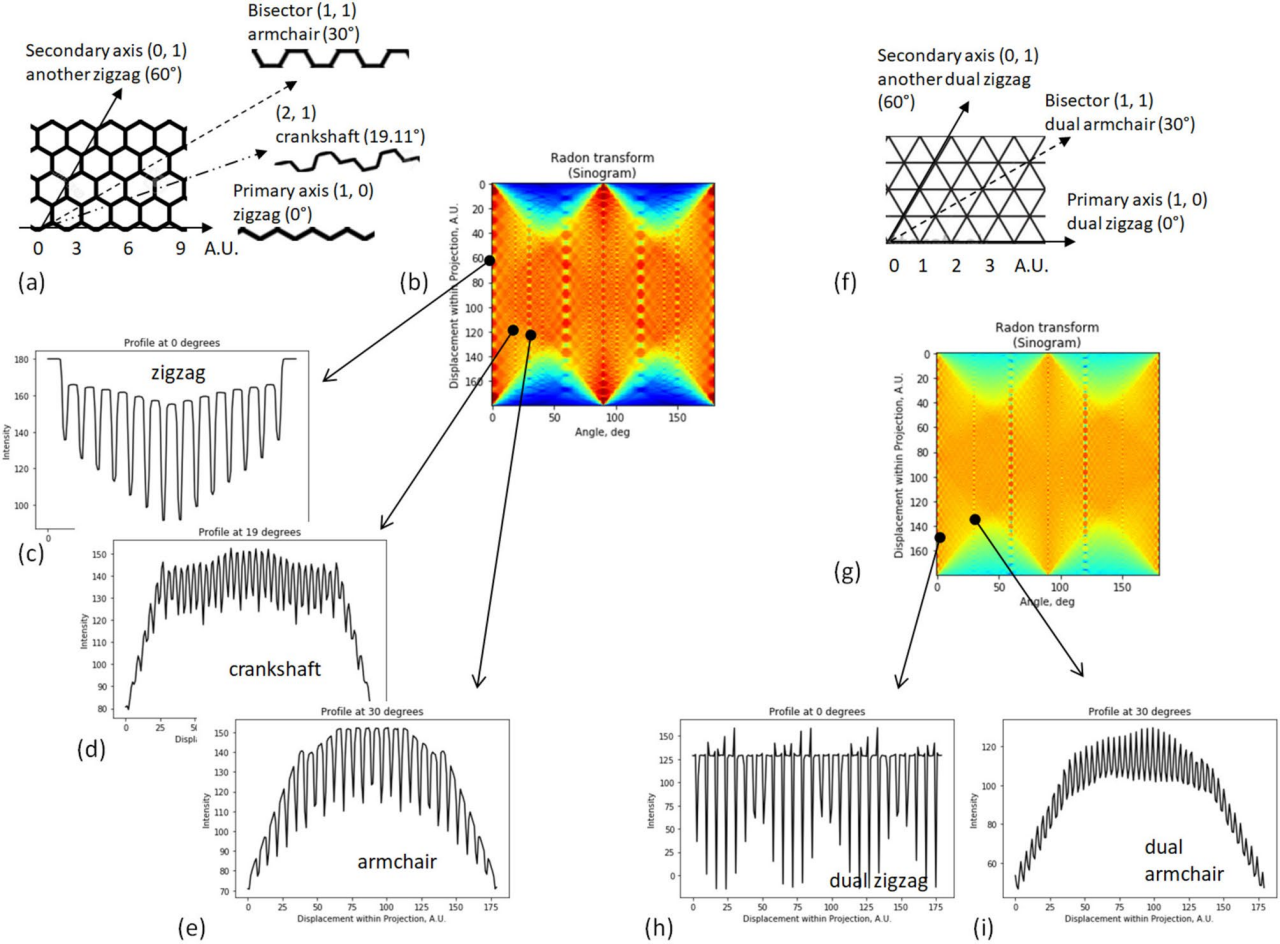
Radon transform of hexagonal and triangular grids. (**a**) Hexagonal grid {6, 3} and its significant directions. (**b**) Sinogram of Radon transform of {6, 3}. (**c**–**e**) Profiles along three static directions (their names within each plot). (**f**) Triangular grid {3, 6}. (**g**) Its sinogram. (**h**,**i**) Profiles along two static directions. Due to the symmetry, the picture is repeated after 60°.

Together with the gratings, we consider three regular grids (triangular, square, and hexagonal), the graphical equivalents of three regular tessellations existing in the three-dimensional space^51^. The Radon transforms of four periodic structures are shown in Figs. 2, 3 in the following order: the line grating, the square grid {4, 4}, the hexagonal grid {6, 3}, and the triangular grid {3, 6}. (Note that the basic meaning of the Schläfli symbol {*p*, *q*} is that there are *q* regular *p*-gons at each vertex^51^). Due to the rotational symmetry, the static directions are repeated after 180° in the line grating, after 90° in the square grid, and after 60° in triangular and hexagonal grids; this can be seen in Figs. 2b, e, 3b, g.

Note that the only static direction of the grating is its axis, see the sinogram in Fig. 2b. However in Figs. 2e, 3b,g, other periodic structures can be also observed at other angles.

In the sinogram Fig. 2e, periodic structures of {4, 4} can be recognized at the following angles: the coordinate axes of the Cartesian system and the bisectors between them. These directions can be expressed in the rectangular coordinates as (1, 0) and (1, 1), see Fig. 2d. The diagonal direction is shown by the dashed line.

The static directions of {6, 3} in two quadrants are the axes 0°, 60°, and 120°, plus additionally discovered bisector angles 30°, 90°, and 150°, see Fig. 3b. The numerical coordinates are also provided in Fig. 3a, although in the skew system. Recall that in the nanoparticles, these directions are known as the “zigzag” and “armchair”^62,63^; namely, the former triplet of angles corresponds to the zigzag directions, the latter to the armchair, see Fig. 3a, where the additional direction is shown by the dashed line. The symmetric structure of {6, 3} is not only repeated after 60° but also “reflected” around the bisector, as can be seen in Fig. 3a. Therefore, it is enough to mention the directions between the primary axis and the bisector, i.e., within the angular range of 30°. Note that the periods of the cross-sections of the sinogram (see Fig. 3b) at two mentioned static directions are different; besides, the visual contrast at the armchair direction is less than at the zigzag, see Fig. 3c–e.

Together with these two, an extra static direction (“crankshaft”) can be recognized in {6, 3} at the angle arctan(√3/5) = 19.11°. The visual contrast at this extra direction is even less than in the armchair direction.

Similarly to the hexagonal grid, there are the static directions at 0° and 30° in the triangular grid, see Fig. 3f–i. Recalling the duality relation^51^ between these grids {3, 6} and {6, 3}, the static directions of the triangular grid {3, 6} can be referred to as dual zigzag and dual armchair. A dual crankshaft was not clearly recognized, though.

The periods of the moiré patterns along two static directions of the triangular grid {3, 6} are *a*_3_√3/2 and *a*_3_/2, where *a*_3_ is the side of the triangle. In the hexagonal grid, the periods along the zigzag, crankshaft, and armchair directions are 1.5*a*_6_, 1.5*a*_6_√7 = 0.57*a*_6_, and, 0.5 *a*_6_√3 = 0.87*a*_6_, resp., where *a*_6_ is the side of the hexagon (the bond length in graphene is 0.142 nm^64,65^).

Let’s estimate the visibility of the static patterns in the hexagonal grid {6, 3} by Michelson’s definition^66^. In the cross-sections of the sinogram of the hexagonal grid, the width of the pulse at the minimum is equal to √3/8*a*_6_ = 0.22*a*_6_ in the zigzag case, to 0.5*a*_6_ in the crankshaft case, and to the width of the line in the armchair case. The corresponding opening ratios are 0.33, 0.38, and 0.14, resp. (for the line width of 3 pixels and the side of the hexagon of 25 pixels). The calculated values of the Michelson contrast at three directions are 13%, 6%, and 46%; these values were confirmed in the numerical simulation. However, despite the lower calculated contrast, the subjective visibility of the static zigzag pattern is highest of three static directions; this is probably because of very narrow pulses of the armchair case (and thus, the lowest opening ratio). The crankshaft case is definitely least visible and it was difficult to observe it experimentally; nevertheless, formally it exists as the third static direction of the hexagonal grid.

The primary (axial) static directions of the square grid are 0° and 90° (abscissa and ordinate); the moiré patterns at these directions have the highest contrast. The static directions with the highest contrast in triangular and hexagonal grids are 0°, 60°, and 120° (the zigzag directions of the hexagonal grid). Besides, there are the additional static directions with lower contrast at ± 45° (diagonal) in the square grid, and 30°, 90°, and 150° (angle bisector between the zigzag axes) in the triangular and hexagonal grids. At the bisector direction in the hexagonal grid {6, 3}, the visual effect is weaker than at the bisector directions in the triangular or square grids {3, 6}, {4, 4}. This is because of the approximated lines of {6, 3} which are explained in Supplementary Note S2 online. Due to the low opening ratio of dashes, the expected contrast is less than in the zigzag case. Moreover, in the hexagonal grid, an extra crankshaft direction with the lowest contrast was identified at ± 19.11°, 60° ± 19.11°, and 120° ± 19.11°.

### Moiré patterns in moving (sliding) grids

Consider the planar movement of a grid along its static directions. When an object (particularly, made of approximated lines, a couple of examples of which are shown in Fig. 1g,h) moves along the axis, the moiré patterns have a linear structure (i.e. consists of parallel lines), see Supplementary Video S2 online.

Only one line pattern can remain static in its place while grid moves. This particular moiré pattern is formed by one pair of elementary line gratings (or approximate line gratings) from which the grids are composed; all other geometric elements of the moving grid are averaged to the uniform gray field.

**Figure 4:**
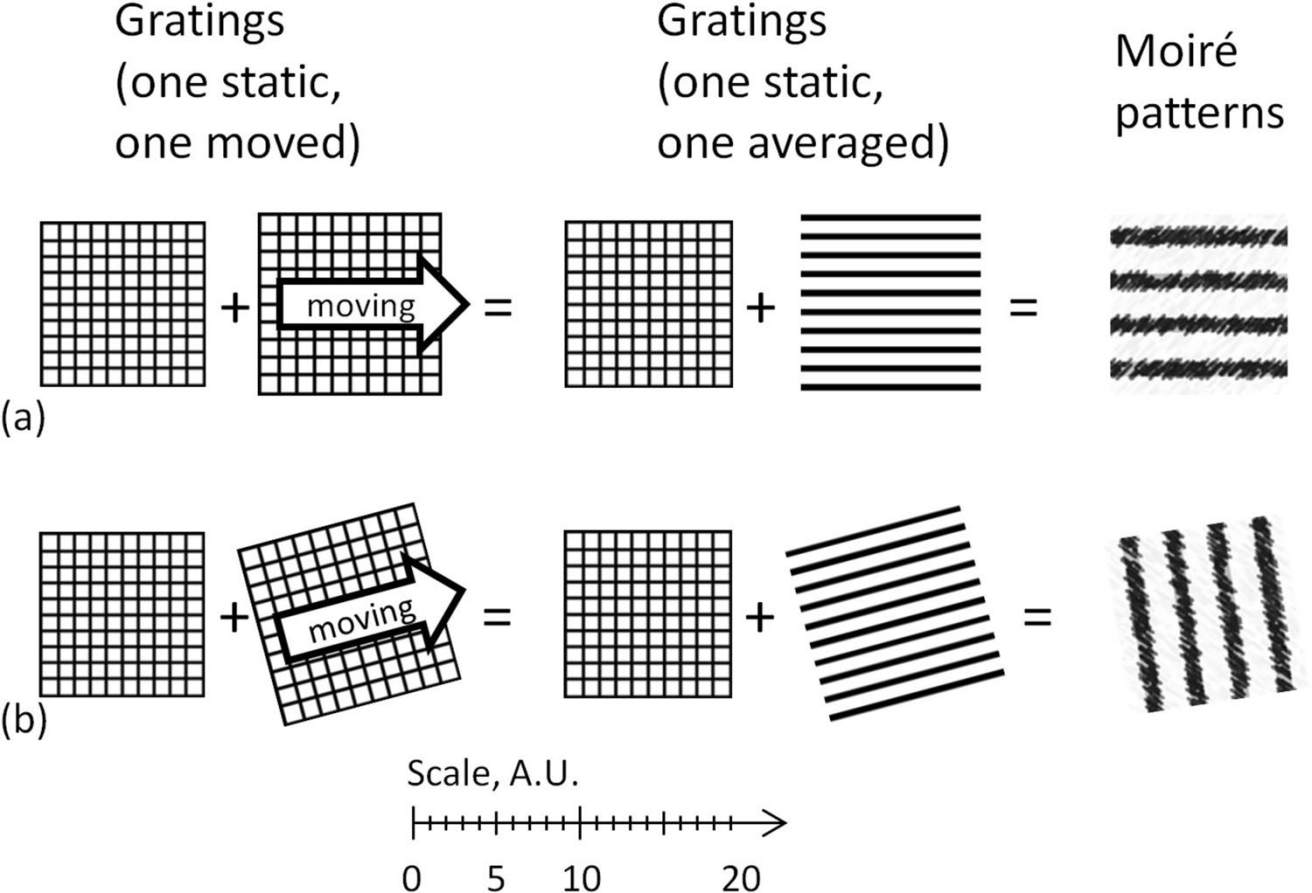
(**a**) The moiré patterns in identical static + sliding grids of identical orientations. (**b**) The moiré patterns in non-identical static + sliding grids with non-identical twisted orientations. The scale is common for (**a**,**b**).

Therefore, the moiré effect in a static grid overlapped with another grid moving along its static direction is equivalent to the moiré effect in that grid overlapped with an equivalent (static) grating (which is the moving grid averaged along the static direction). Examples are shown in Fig. 4 for the parallel and twisted gratings.

In Fig. 4a (identically oriented grids of different periods), the axis of the moiré patterns coincides with the translation vector. However, in twisted gratings, the picture depends on the relationship between parameters of gratings (for details, refer to Supplementary Note S1 online), so as the moiré axis might considerably deviate from the translation vector, and can be almost perpendicular to it, see Fig. 4b, Supplementary Videos S3, S4 online. This is the main reason for the visual unexpectedness of the moiré effect in the sliding grids.

### Static directions in coplanar gratings

For the line moiré patterns to remain in place without a drift, the phase difference between the phases of the gratings Δ*φ* = *φ*_1_ − *φ*_2_ (each by Eq. (1)) must be constant (see Supplementary Note S1 online).

For the gratings of arbitrary periods with the collinear axes and the collinear translation vector, the visual picture of the patterns does not change, as in Fig. 4a and in Supplementary Fig. S3 of Supplementary Note S1 online. In this simple case of the moiré effect, the only two static directions are the axes of either grating (coinciding axes).

**Figure 5:**
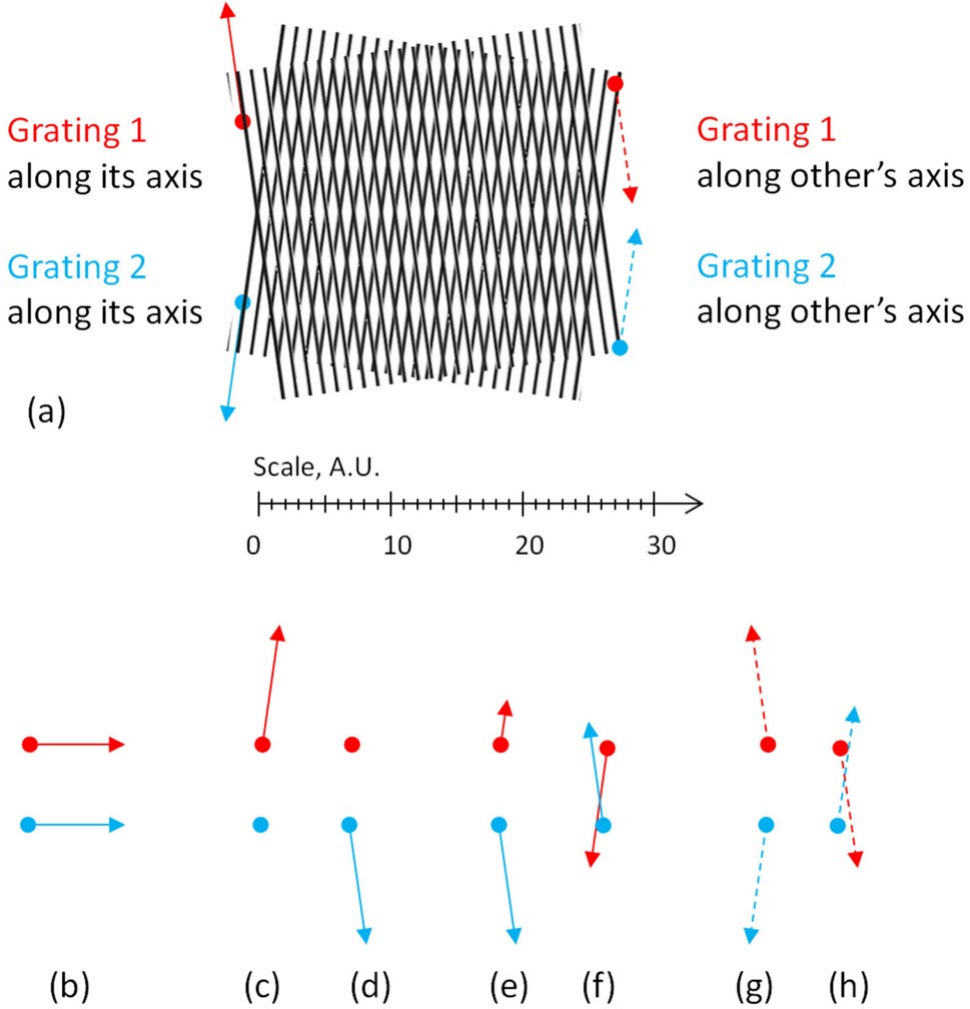
(**a**) Moiré patterns in gratings (static directions of each grating are shown by thin arrows). (**b**–**h**) Static cases of the moiré patterns in 2 gratings. In (**e**,**f**), two velocities are not necessarily equal. In (**c**–**f**), the gratings move along own axes; in (**g**,**h**), the gratings move along other’s axes.

The case of the gratings with non-collinear axes shown in Fig. 4b is richer. For instance, if the axes of two identical coplanar gratings symmetrically deviate from the vertical (twisted around the *x*-axis by equal positive and negative angles, but one clock-wise, another counter clock-wise), the axis of the patterns is exactly horizontal as shown in Fig. 5a.

The condition of the constant phase means that either each grating moves along its own axis (when *φ*_1_ = const and *φ*_2_ = const) or it’s a coordinated movement of both gratings with the constant difference (when *φ*_1 _− *φ*_2_ = const). The latter option can be satisfied in two cases: when both gratings move as a solid structure (a trivial case) or when each grating moves along the other axis in converging/diverging directions with equal |Δ*φ*_*i*_| but opposite signs of each particular Δ*φ*_*i*_ (a synchronized coherent movement). Correspondingly, the movements which do not affect the visual picture of the moiré patterns are as follows:

- Both gratings along the moiré axis together (a trivial collective motion as a solid body), see Fig. 5b.
- Each grating along its axis (its static direction) as shown by arrows in Fig. 5a, with no regard to a possible movement of another grating, and the visual picture is unchanged. This is an individual motion with an arbitrary speed; see Fig. 5c,d, and Supplementary Video S2 online. Such independent movements of the gratings in converging/diverging directions can be combined together, probably with different speeds, see Fig. 5e,f.
- The synchronized (coherent) motion of both gratings along others’ axes with equal speeds, see Fig. 5g,h, and Supplementary Video S5 online.

However, if a grating moves along the other’s axis with a small twist angle, the moiré patterns drift; see Supplementary Video S1 online.

It is not uninteresting to find that when a grating moves along its axis, the static pattern could be at a large angle (almost perpendicular) to the direction of movement. This is a paradoxical, unexpected feature of the individual movements, especially counterintuitive when the movement of the grating can be clearly seen in the beginning and at the end of Supplementary Videos S2–S4, as well as in Supplementary Video S5 online. Note that in the recorded supplementary videos, the axis of the moiré patterns is (a) not horizontal due to the layout of the experiment and (b) somehow deviated from the expected direction of the coplanar case due to the distance effect, which will be described below.

### Static moiré patterns in sliding coplanar gratings and regular grids

**Figure 6:**
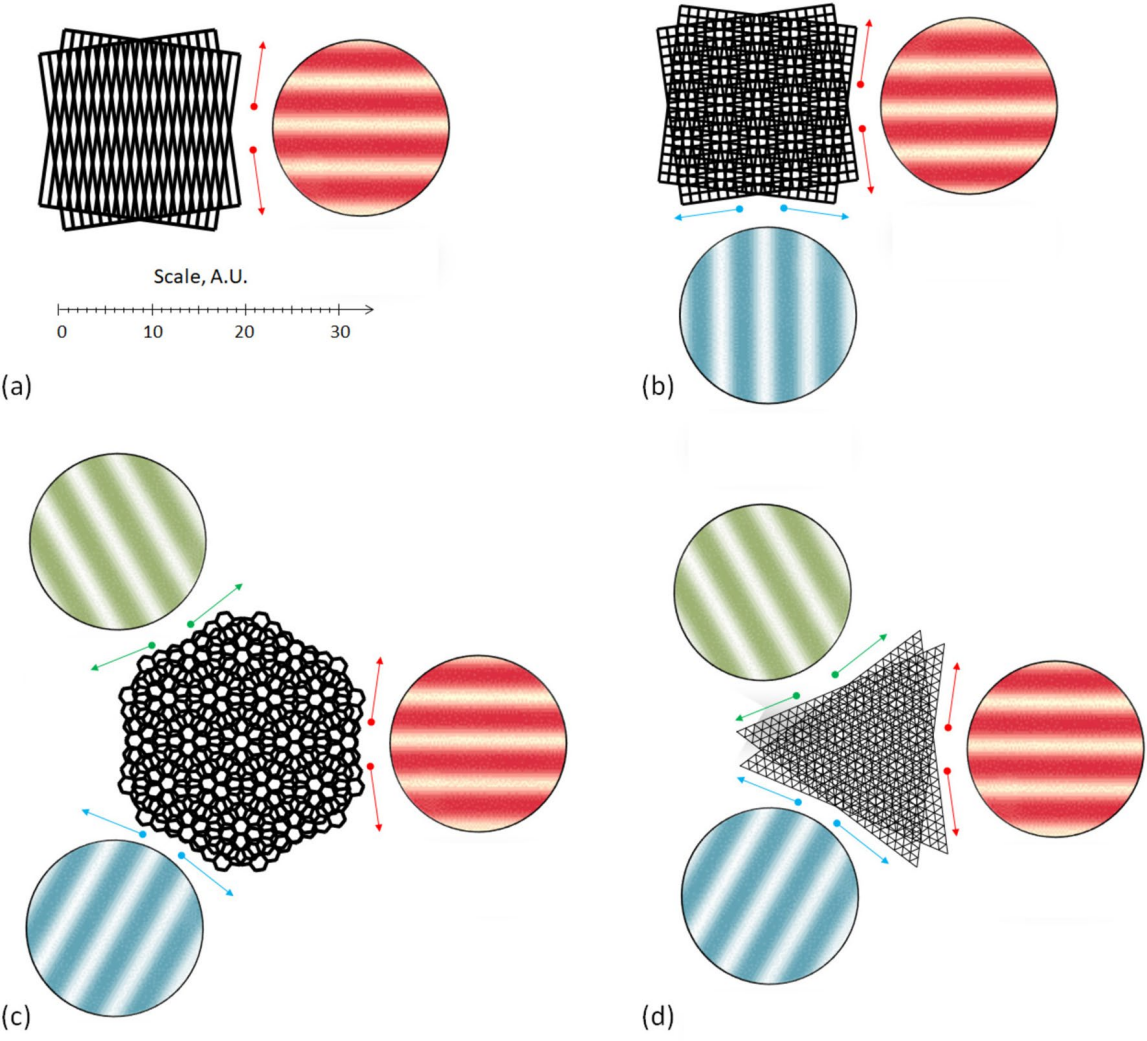
Moiré effect with the highest contrast (**a**) in line grating and (**b**–**d**) in regular grids. Conditional pseudo colors “red”, “green”, etc. show static patterns and static directions. The scale is common for (**a**–**d**).

If the twisted axes are symmetric around the abscissa, the axis of the moiré patterns is exactly horizontal. For instance, in Fig. 6a, the axis of the static moiré patterns is horizontal, while in Fig. 6b the two static axes are horizontal and vertical. The static moiré patterns in Fig. 6 are painted in pseudo colors and encircled; the static directions of the grids are shown by correspondingly colored arrows. The static moiré patterns in moving objects are shown in Supplementary Videos S2–S9 online.

The square grid is a combination (superimposition) of two orthogonal gratings, as explained in Supplementary Note S2 online. Therefore, there are four line structures in two overlapped square grids and thus, four static directions of the moiré patterns in them (each elementary grating along its axis), see Fig. 6b, Supplementary Videos S3, S4 online.

The hexagonal grid {6, 3} is a superimposition of three approximate gratings; refer to Supplementary Note S2 online. Therefore, there are six individual static directions in the hexagonal grid along the approximated zigzag lines, see Fig. 6c. These patterns have the highest contrast. Together with six armchair directions, 12 static directions of the moiré patterns with a relatively high contrast can be identified in the hexagonal grid. However, there are six extra directions (crankshaft) with the lowest contrast in each grid. Therefore, the total theoretical number of the static directions of the moiré patterns in the superimposed hexagonal grids is 24. The visual examples of the static moiré patterns in the hexagonal grids moving along the static directions are shown in computer-generated Supplementary Videos S6, S7 online.

Similarly to the square grid, the triangular grid {3, 6} can be thought of as a combination of three line gratings with a proper spatial phase, see Fig. 6d, Supplementary Note S2 online. Correspondingly, there are six line structures in two overlapped triangular grids. Therefore, there are six static directions of the moiré patterns the highest contrast along the dual zigzag direction of each grid. Together with six dual armchair directions, 12 static directions of the moiré patterns with a relatively high contrast can be identified in the triangular grid. For visual illustrations, refer to Supplementary Videos S8,S9 online.

Supplementary Videos S6–S9 are made at armchair and zigzag directions with the small twist angle between the grids. Note that there is a noticeable angle between the axis of the moiré patterns and the axes of the gratings in Supplementary Videos S6, S8. Along with the computer-generated Supplementary Videos S5–S9, the recorded Supplementary Videos S1–S4 are provided.

### Non-coplanar gratings in motion

When the twist angle between the gratings is zero, the axes of the gratings and of the moiré patterns are collinear. With a non-zero twist angle, the orientation of the moiré patterns depends on the distances (to the observer, between the gratings); this is illustrated in^53^. It means that with all other parameters equal except for the distances, the patterns rotate and magnify/de-magnify, refer to Supplementary Eqs. (S14), (S15) online. See also Fig. 4, Supplementary Fig. S3, and Supplementary Videos S3, S4 online.

In the case of the lateral motion along the static directions combined with the distance effects, the behavior of the moiré patterns becomes complicated. For instance, although the static directions in the gratings remain indeed the same, the visible patterns rotate depending on the gap and the distance. The picture of the moiré patterns might appear entirely different from the moiré patterns in coplanar grids. This can be observed in the recorded Supplementary Videos S2–S4 online.

In the case of the longitudinal motion (an observer approaches/comes off to/from the gratings), the moiré patterns rotate and their period also changes. This is a distance effect described in Supplementary Note S1 online, particularly, by Supplementary Eqs. (S14), (S15).

**Figure 7:**
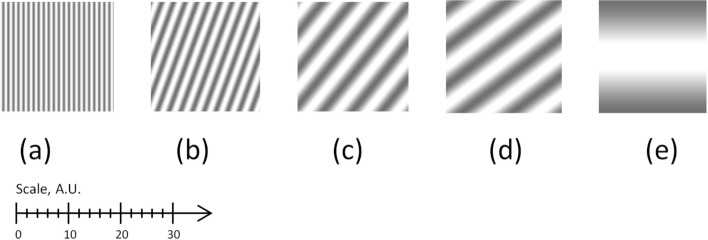
Moiré patterns in non-coplanar gratings at different distances: (**a**) 0, (**b**) 30 cm, (**c**) 80 cm, (**d**) 130 cm, (**e**) ∞.

Theoretically, at a short (infinitesimal) distance, the axis of the patterns in identical gratings is at the angle *α*; at a long (infinite) distance, it is orthogonal to the short-distance patterns. Therefore in principle, the axis of the patterns can vary between 0 and π/2, see Fig. 7. Practically, it is within the angular range 45°–50°.

Therefore, in a non-coplanar motion with an unspecified distance, the orientation of the axis of the moiré patterns is virtually unpredictable, see Fig. 7 for the simulated patterns at short, middle, and long distances (the angle between the axes of non-collinear gratings is 10°, the gap between them is 10 cm).

In terms of the angle and period, the visual picture of the moiré patterns in the non-coplanar gratings observed from the infinite distance (Fig. 7e) is the same as that for the coplanar gratings shown in Fig. 1a (where the distance does not affect the angle at all).

## Discussion

When an object moves, the visual or photographed picture is blurred; this corresponds to our common-sense expectation for a picture to be blurred due to motion. However in the movement along the static directions (especially in the presence of the distance effect), the vertical or slanted patterns can be unexpectedly kept, even if we can clearly see the movement of the object. In such case, the overall picture of the moiré patterns and the movement may look like a strange counter-intuitive puzzle. The common sense obviously tells us that no vertical line can withstand against a fast horizontal move; nevertheless, we can see it.

Such an “mismatch” between the visual picture and common sense can be used positively in security applications, especially when the geometry of an object is not obvious (e.g., a hidden or dashed pattern), like at the armchair axis and especially at the crankshaft axis of the hexagonal grid (if, indeed, the contrast of that case could be improved).

From the geometrical point of view, the zigzag and armchair directions of the hexagonal grid can be considered as skew analogs of the ordinate and the diagonal of the square grid. The corresponding coordinates are (0, 1) and (1, 1) on both orthogonal and skew grids. In theory, the static angles in the triangular and hexagonal grids might exist at higher values of the integer coordinates, e.g., at (1, 3), (3, 2), etc. Practically, however, the low contrast prevents observing the moiré patterns at these angles. In the square grid, the moiré patterns were observed at rational angles with *m* and *n* up to 5, refer to^67^. In this paper, we observed the crankshaft direction at 19.11° with the skew coordinates (2, 1), and one coordinate was larger than 1, although the visual contrast was very low.

In respect to the moiré effect, the dual grids were briefly considered in^68^. Namely, it is found that the structure and the period of the moiré patterns in the dual tessellations are identical. However, together with the main pattern (which repeats the structure of the grid), there are additional series of the patterns in the triangular grid {3, 6}; they have the same period but the different orientation and phase accompanied by the lower contrast (most probably, these are caused by the structure of {3, 6} consisting of “straight” and “mirrored” triangles. In order not to be confused by such a complicated structure of not identically oriented series of the moiré patterns, additional care should be taken when considering the moiré patterns in the overlapped triangular grids. From this point of view, the moiré patterns in the hexagonal grid look simpler (they just reproduce the grids without side effects).

Semi-regular tessellations and non-planar cases can bring a variety of options; however other structures (those which cannot be represented as combinations of the gratings, even approximate) may not have static directions at all.

We consider the moiré effect in an object and its twisted or resized copy. However, the static patterns in arbitrary combinations of various objects are not impossible.

The phase does not affect the static directions, although the structure of the tessellation may be affected, recall Supplementary Fig. S6b in Supplementary Note S2 online.

In practical applications, the static patterns in the moving gratings can be used for the detection of the periodic structure along a certain direction, e.g., in the counterfeit detection. In this case, the first grid (say, complicated and not obviously recognized) is printed on a valued document, while the second grid (say, simple but invisible for a user) is built-in into a verification device. The direction of movement and the distance can be predefined or coded in the document by an independent method. The moiré patterns in the device remain static if only a specimen moves along a certain (secret) direction at a certain (hidden) gap. To detect the static patterns, a high-speed camera for fast-moving objects is unnecessary. The verification device moves over the document along the known direction, and once the static moiré pattern is recognized, the validity certificate (message) is issued.

Another example could be some dynamic artwork or a visual attraction. For example, when a car/train with the meshed windows passes near a static grating, the moiré patterns will appear in the window, and their direction could be unexpectedly almost perpendicular to the direction of the movement.

## Methods

**Figure 8:**
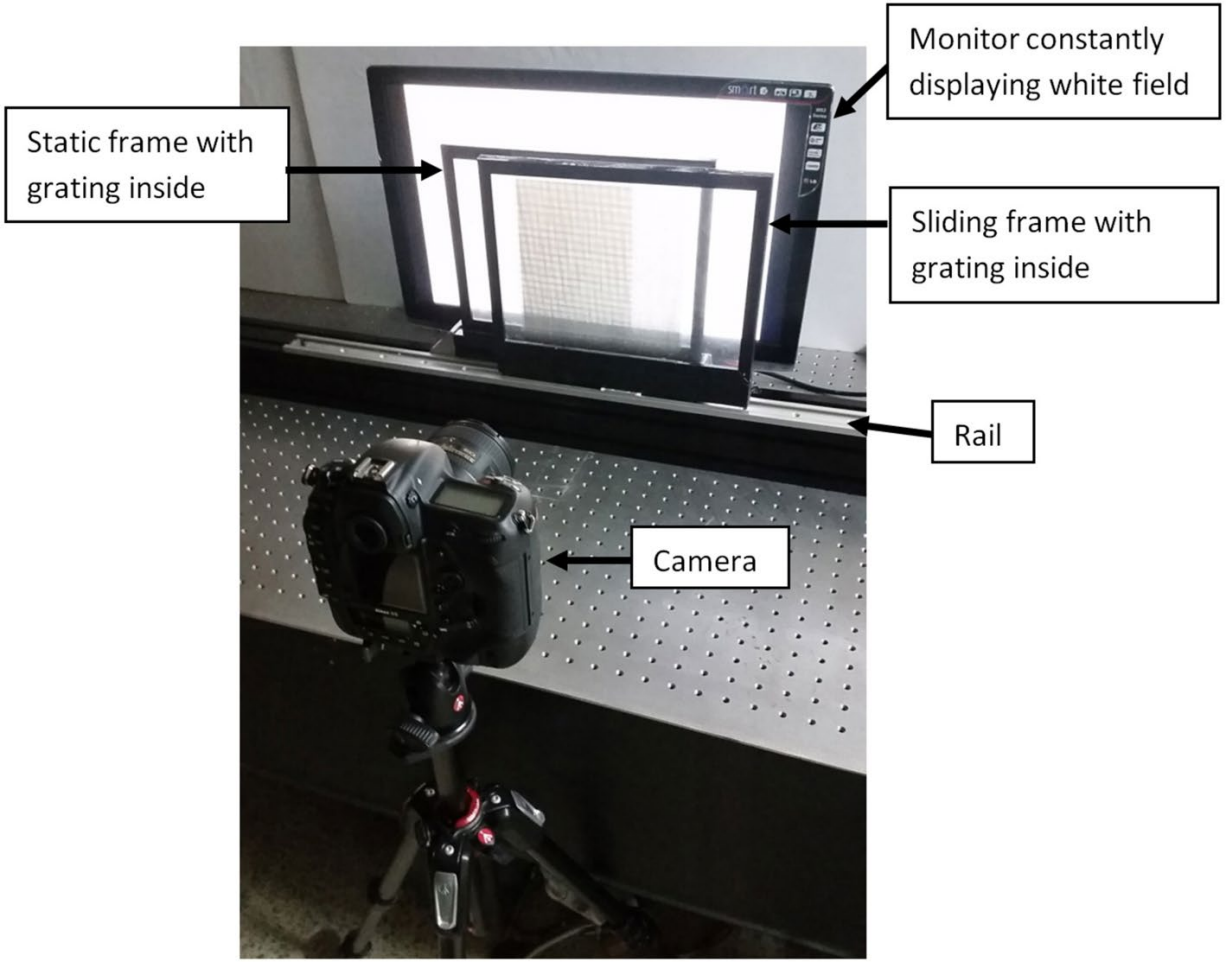
Photograph of experimental setup.

In the physical experiment, we used the following equipment, the photographic camera Nikon D5, two rectangular plastic frames of the A4 size (one static, one sliding horizontally along the rail), and a uniform backlight (a computer monitor constantly displaying a white field). The gratings were printed on transparencies of the A4 size by a laser printer and installed in the frames between thin glasses (thickness about 1 mm). The air gap between the sliding transparencies was 4 cm. As the grating in the moving frame slides along a static direction, the static moiré patterns can be observed and photographed. Experimental photographs were taken from the distances between 25 and 50 cm. The experimental setup (the overall size 100 cm × 40 cm,) is shown in Fig. 8.

In the computer simulation, we used the images of the grids with the horizontally oriented primary axis combined with the images of the twisted grids. To simulate a high-speed movement, the motion blur was applied along the direction of motion. The images of the static and sliding grids were overlapped, and the moiré patterns appeared; this way, each phase of the movement (one snapshot of the movie) was prepared. Then, the set of prepared snapshots was merged into a single gif-file and further arranged (cut length, etc.) with using free online services at ezgif.com-gif-maker, onlineconverter.com, and the like.

## Supplementary information

Supplementary Information

Supplementary Notes S1 & S2

Supplementary Video S1

Supplementary Video S2

Supplementary Video S3

Supplementary Video S4

Supplementary Video S5

Supplementary Video S6

Supplementary Video S7

Supplementary Video S8

Supplementary Video S9

## Data Availability

Authors can confirm that all relevant data are included in the paper and/or its Supplementary information files.
