# Author Correction: Static moiré patterns in moving grids

**DOI:** 10.1038/s41598-022-06349-7

**Published:** 2022-02-02

**Authors:** Vladimir Saveljev, Jaisoon Kim, Jung‑Young Son, Yongsuk Kim, Gwanghee Heo

**Affiliations:** 1 Public Safety Research Center, Konyang University, Nonsan, Chungcheongnam-do 32992 Republic of Korea; 2 Department of Physics, Myongji University, Yongin, Gyeonggi-do 17058 Republic of Korea; 3 Department of Medical IT Engineering, Konyang University, Daejeon, 35365 Republic of Korea; 4 Department of Civil and Environmental Engineering, Konyang University, Nonsan, Chungcheongnam-do 32992 Republic of Korea

Correction to: *Scientific Reports* 10.1038/s41598-020-70427-x, published online 02 September 2020

The Supplementary Video S1 published with this Article contained an error where the vector “Direction of move” deviated from its horizontal line. The original Supplementary Video S1 is provided below.

This error has now been corrected in the Supplementary Video S1 file that accompanies the original Article.

## Supplementary Information

Supplementary Video S1.

