# Supplementary material for: Static moiré patterns in moving grids
Source: Sci Rep. 2020 Sep 2;10:14414. doi: 10.1038/s41598-020-70427-x (PMC7468115; doi:10.1038/s41598-020-70427-x)
Supplement: Supplementary file 1 — Supplementary Information [file 41598_2020_70427_MOESM1_ESM.docx]

Supplementary Information Guide

Static moiré patterns in moving grids

V. Saveljev, J. Kim, J.-Y. Son, Y. Kim, and G. Heo

Supplementary Information

This file contains two Supplementary Notes: S1) about the static moiré effect and the condition of the constant phase, S2) about the regular 2D grids built from 1D gratings. These Notes include 21 Equations and 8 Figures.

Supplementary Video

Supplementary Videos S1 – S4 are recorded by a photo camera. Supplementary Videos S5 – S9 are computer-generated.

1) File Name: Supplementary Video S1

Description: Drifting moiré patterns in twisted line gratings, one sliding along other’s axis, recorded video.

2) File Name: Supplementary Video S2

Description: Static moiré patterns in twisted line gratings, one sliding along own axis, recorded video.

3) File Name: Supplementary Video S3

Description: Static moiré patterns in sized square grids, one sliding along own axis (abscissa), recorded video.

4) File Name: Supplementary Video S4

Description: Static moiré patterns in twisted square grids, one sliding along own axis (abscissa), recorded video.

5) File Name: Supplementary Video S5

Description: Static moiré patterns in twisted line gratings, both sliding along own axis.

6) File Name: Supplementary Video S6

Description: Static moiré patterns in twisted hexagonal grids, one sliding along own axis (zigzag).

7) File Name: Supplementary Video S7

Description: Static moiré patterns in twisted hexagonal grids, one sliding along own axis (armchair).

8) File Name: Supplementary Video S8

Description: Static moiré patterns in twisted triangular grids, one sliding along own axis (dual zigzag).

9) File Name: Supplementary Video S9

Description: Static moiré patterns in twisted triangular grids, one sliding along own axis (dual armchair).
