# Supplementary material for: Static moiré patterns in moving grids
Source: Sci Rep. 2020 Sep 2;10:14414. doi: 10.1038/s41598-020-70427-x (PMC7468115; doi:10.1038/s41598-020-70427-x)
Supplement: Supplementary file 2 — Supplementary Notes S1 & S2 [file 41598_2020_70427_MOESM2_ESM.docx]

Supplementary Information:

Static moiré patterns in moving grids

V. Saveljev, J. Kim, J.-Y. Son, Y. Kim, and G. Heo

Supplementary Note S1

Static moiré effect (no move). Condition of the constant phase

In this Supplementary Note S1, we recall the moiré effect in the static (non-moved) coplanar and non-coplanar line gratings and generalize some of them with special attention to the phase.

**Coplanar gratings**. It is known that the wavevector of the moiré patterns **k***_m_* is a linear combination of the wavevectors of the gratings **k**_1_ and **k**_2_ with the coefficients ±1 (i.e., the sum or difference), whichever of them has the shortest wavevector (falls within the visibility circle) [1], see Supplementary Fig. S1,

$\mathbf{k}_{m}=\mathbf{k}_{1}\pm\mathbf{k}_{2}$ (S1)


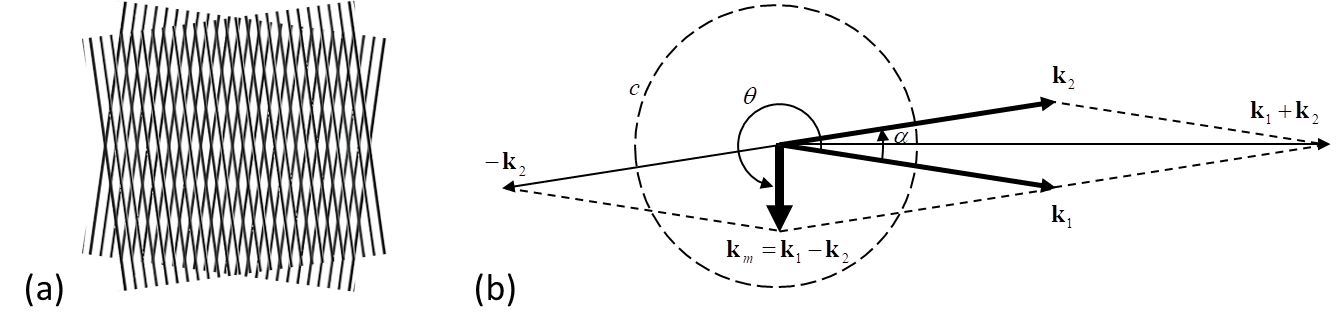


Supplementary Fig. S1. (a) Line gratings, (b) wavevectors. The visibility circle is drawn by the dashed line.

The previous sentence can be considered as the definition of the moiré wavevector. From the law of cosines, the wavenumber of the moiré patterns *k* (scalar, not a vector) is equal to the modulus of the algebraic sum of vectors,

$k_{\pm}=\frac{k_{1}}{\rho}\sqrt{\rho^{2}\pm2\rho\cos\alpha+1}$ (S2)

where *k*_1_, *k*_2_ are the wavenumbers of the gratings, *α* is the angle between the wavevectors, *ρ* is the ratio of wavenumbers (or, equivalently, the periods in the inverse order) defined as follows,

$\rho=\frac{k_{1}}{k_{2}}=\frac{\lambda_{2}}{\lambda_{1}}$ (S3)

By definition [1], the moiré wavenumber is the smaller of two wavenumbers in Supplementary Eq. (S2); in our case, we select the minus sign to get smaller,

$k_{m}=\frac{k_{1}}{\rho}\sqrt{\rho^{2}-2\rho\cos\alpha+1}$ (S4)

The period of the patterns *λ_m_* derived from Supplementary Eq. (S4) will be proportional to *λ*_1_. Therefore, the formula for the period can be rewritten in a special form [2] emphasizing the proportionality of the moiré period to the period of the grating,

$\lambda_{m}=\mu\lambda_{1}$ (S5)

where the coefficient of proportionality (the moiré magnification factor) is

$\mu=\frac{\rho}{\sqrt{\rho^{2}-2\rho\cos\alpha+1}}$ (S6)

Supplementary Eq. (S5) shows how much the period of the grating is magnified, which essentially means a moiré magnifier originally proposed in [3], [4].

Other characteristics of the moiré wavevector (the phase and the direction) are as follows [2],

$\theta_{m}=-\arctan\frac{\sin\alpha}{\rho-\cos\alpha}$ (S7)

$\phi_{m}=\phi_{1}-\phi_{2}$ (S8)

For the identical gratings, the direction of the moiré wavevector derived from Supplementary Eq. (S7) is the half-angle between the first wavevector and the negative second wavevector.

Supplementary Equations (S4), (S7), (S8) comprise the full description for the wavevector (wavenumber, phase, and orientation). From these equations, the following necessary conditions for the pattern to remain unchanged are as follows,

$\rho=const$ (S9)

$\alpha=const$ (S10)

$\phi_{1}-\phi_{2}=const$ (S11)

In Supplementary Eq. (S9), the value of *ρ* is constants due to the mechanical properties of solid gratings; the angle *α* in Supplementary Eq. (S10) is constant because the directions of the axes are fixed mechanically; while the third condition Supplementary Eq. (S11) means that either both *φ*_1_ = const, *φ*_2_ = const (each grating moves along its own axis) or a coordinated movement of both gratings with the constant difference *φ*_1_ - *φ*_2_ = const.

The above formulas for the characteristics of the moiré patterns with the zero phase are not unknown in the literature; similar formulas can be found in [1], [5] - [8] and elsewhere. We provide them here with the phase, which is essential for the current content.

**Non-coplanar gratings in parallel planes**. In the non-coplanar case (the gap *d* between the parallel planes of the gratings is not zero), the picture may look complicated [1], [10], even if the gratings are identical and their planes are parallel. In parallel non-coplanar gratings [11], the period of the second grating projected onto the plane of the first one is

$\lambda_{2}'=\frac{\rho}{s}\lambda_{1}$ (S12)

where *ρ* is defined by Supplementary Eq. (S3) and the geometric characteristic of the layout of the gratings is

$s=1+d/z$ (S13)

Then, substitute $k_{2}'=k_{1}\cdot s/\rho$ by Supplementary Eq. (S12) instead of *k*_2_, into the law of cosines for the wavevectors **k**_1_ and **k**_2_, we can obtain the generalized Supplementary Eq. (S6) as follows,

$\mu=\frac{1}{\sqrt{\chi^{2}-2\chi\cos\alpha+1}}$ (S14)

where the coefficient *χ* = *s*/*ρ*.

Furthermore, the Supplementary Eq. (S7) generalized to the non-coplanar case is,

$\tan\theta=\frac{-\sin\alpha}{\frac{1}{\chi}-\cos\alpha}$ (S15)

Based on Supplementary Eqs. (S14), (S15), the corresponding *χ* can be found so as the visual characteristics (the magnification or the angle) remain the same for any *α*), although the values *μ* and *θ* are controlled separately and thus, these values of *χ* are different. Namely, to maintain the same magnification, the value of *χ* must be

$\chi_{\mu}=\cos\alpha\pm\sqrt{\cos^{2}\alpha-1+\frac{1}{\mu^{2}}}$ (S16)

but to keep the angle, it must be

$\chi_{\theta}=\frac{1}{\cos\alpha-\frac{\sin\alpha}{\tan\theta}}$ (S17)

Therefore, even if the magnification and orientation are not completely interchangeable, their influence can be taken into account by Supplementary Eqs, (S16), (S17).

In the case of the identical gratings, Supplementary Eqs. (S14), (S15) are simplified,

$\mu_{||}=\frac{1}{\sqrt{s^{2}-2s\cos\alpha+1}}$ (S18)

$\tan\theta_{||}=\frac{-\sin\alpha}{\frac{1}{s}-\cos\alpha}$ (S19)

A graphical illustration of the above formulas is shown in Supplementary Fig. S2.

1. (b)

Supplementary Fig. S2. Characteristics of moiré patterns in non-coplanar gratings: (a) magnification factor, (b) rotation angle.

We may consider the non-identical gratings with the collinear axes but different periods, see Supplementary Fig. S3a. In the case of the grating with the periods adjusted according to the distance (*χ* = 1, i.e., *ρ* = 1 + *d*/*z*), see [2],

$\mu_{1}=\frac{1}{2\sin\alpha/2}$ (S20)

$\tan\theta_{1}=-\frac{1}{\tan\alpha/2}$ (S21)

The moiré effect in the twisted identical gratings is illustrated in Supplementary Fig. S3b.

(a)
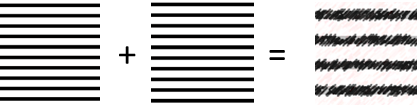


(b)
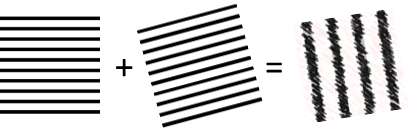


Supplementary Fig. S3. (a) Overlapped non-identical identically oriented gratings produce the moiré effect similar to twisted gratings. (b) Overlapped non-identically oriented (twisted) identical gratings.

Here, we have to point out the noticeable difference of the moiré effect in the identical and in the non-identical gratings. In the former case, all three axes are collinear; but in the latter case, the axis of the moiré patterns is almost perpendicular to the axes of the gratings, as in [9]. This interesting feature is caused by geometry.

Supplementary Note S2

Regular Grids as Superimpositions of Line Gratings

In some cases, a periodic 2D structure can be considered as a superposition of several families of parallel equidistant lines (line gratings). For example, the regular square and triangular grids can be thought of as superpositions of 2 and 3 such families at proper angles. However, while the former superposition may have an arbitrary phase (the displacement along the wavevector) of both gratings, as shown in Supplementary Fig. S4a, the latter requires an exact phase of the third grating, see Supplementary Fig. S4b; otherwise, the result would not be the regular triangular grid.


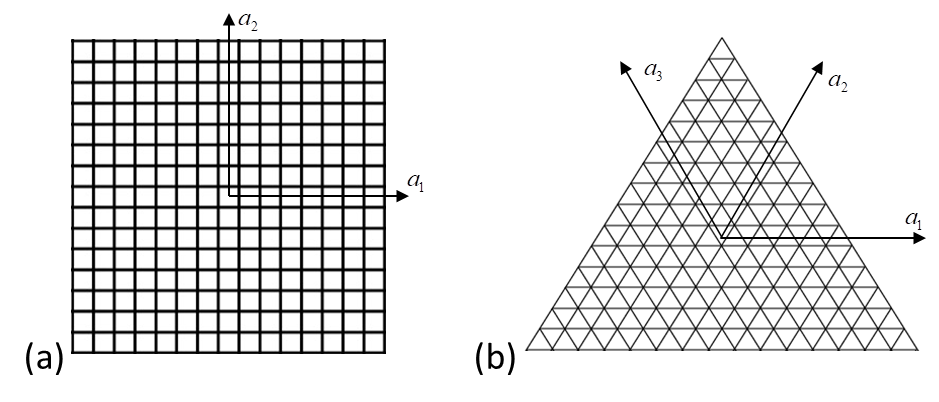


Supplementary Fig. S4. Square and triangular grids and their axes; tessellations {4, 4} and {3, 6}.

The grids shown in Supplementary Fig. S4 can be referred to as the regular tessellations, i.e., the tilings of the Euclidean plane by regular convex polygons. Then, according to [12], the square grid can be denoted as {4, 4} and the triangular grid as {3, 6}; in this notation, the first number denotes the number of polygons at each vertex, the second is the type of the polygon. The third regular tessellation, the hexagonal tessellation {6, 3} (read: three hexagons at each vertex) is shown in Supplementary Fig. S5.


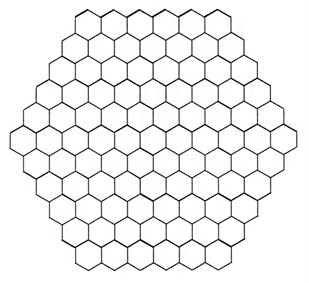


Supplementary Fig. S5. Hexagonal grid, tessellation {6, 3}.

According to [12], the geometric duality means that the center of each polygon is taken as a vertex and these new vertices of adjacent polygons are connected. Therefore, the tessellation {4, 4} is dual to itself, see Supplementary Fig. S6a; the tessellations {6, 3}, {3, 6} are dual to each other, see Supplementary Fig. S6b.


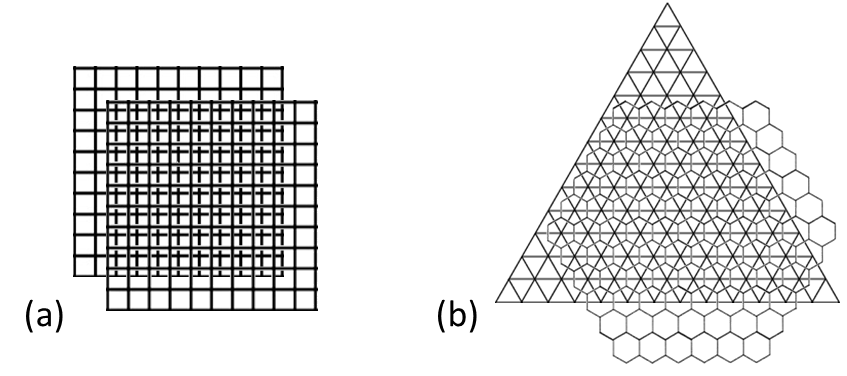


Supplementary Fig. S6. Dual tessellations: (a) {4, 4} and {4, 4}; (b) {3, 6} and {6, 3}.

In contrast to the tessellations {4, 4} and {3, 6}, the tessellation {6, 3} cannot be represented as a combination of the line gratings.

Nevertheless, a hexagonal grid can be built of lines of other kinds. For instance, it can be thought as a combination of either zigzag or dashed lines of a certain particular form (i.e., the angle and the length of zigzag segments, the opening ratio of dashes) with a proper relative position (the phase, the angle, and the distance), as shown in Supplementary Fig. S7. (The opening ratio is the ratio of the length of the segment to the period.) Note that in Supplementary Fig. S7a, each side of the hexagon is comprised of segments of two zigzag lines. In Supplementary Fig. S7a, the zigzag lines are oriented along the axes of the triangular grid (shown in Supplementary Fig. S4b). In Supplementary Fig. S7b, the inclined dashed lines with an opening ratio 1/3 are perpendicular to the zigzag lines of Supplementary Fig. S7a. Proper phases of each line in each family of lines are necessary.


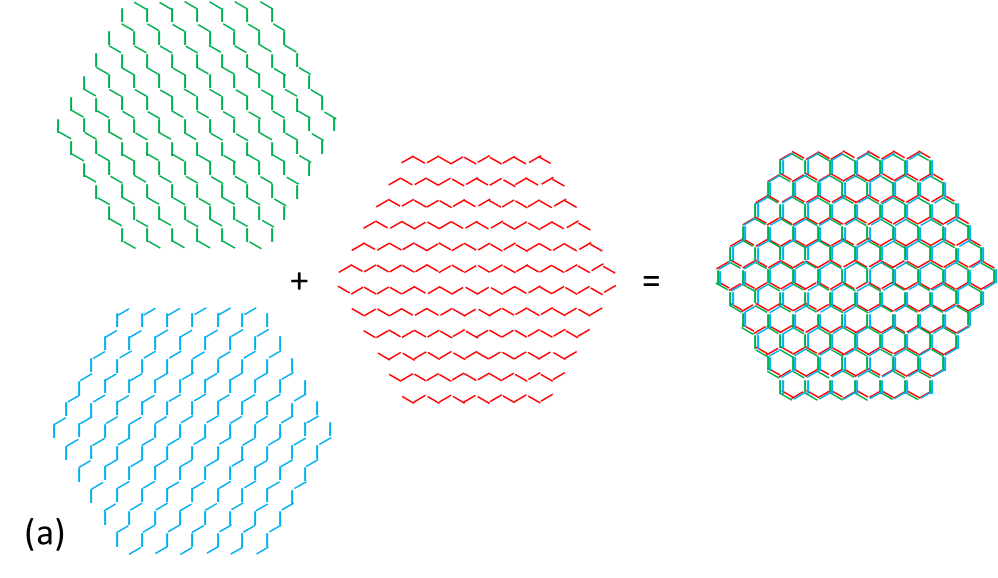


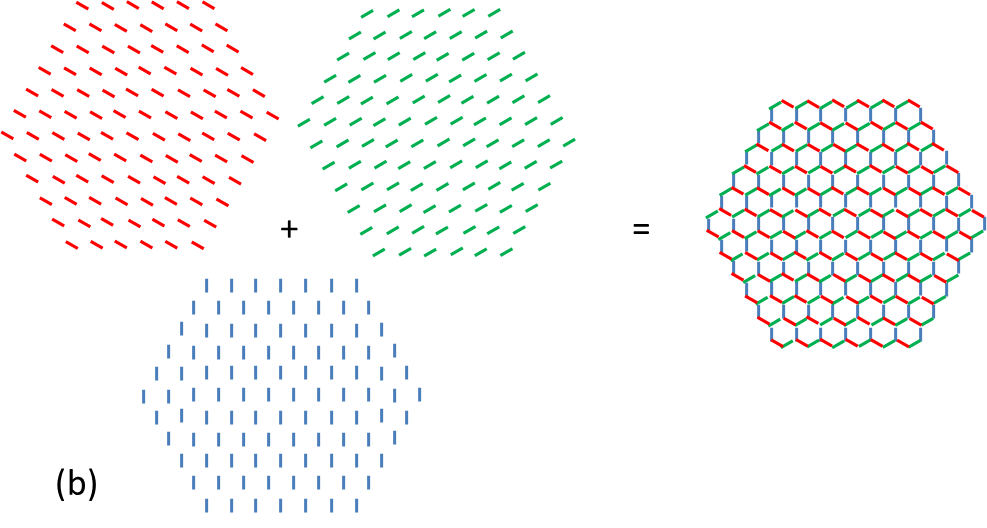


Supplementary Fig. S7. Hexagonal grid as a combination of zigzag and dashed gratings.

The straight lines connecting the centers of the zigzag segments in Supplementary Fig. S7a can be considered as an approximation of the zigzag lines. Three families of such lines may approximate the hexagonal grid, as shown in Supplementary Fig. S8a.


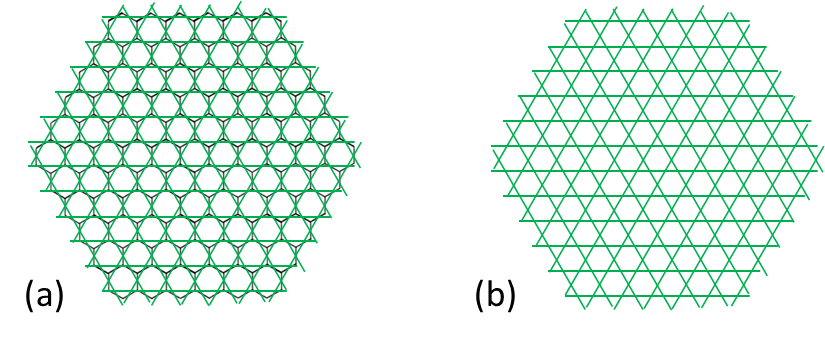


Supplementary Fig. S8. (a) Approximation of zigzag lines in the hexagonal grid by straight lines. (b) Tessellation {3, 6, 3, 6}.

Strictly speaking, the result of such approximation is not a hexagonal grid, rather the semi-regular Archimedean tessellation {3, 6, 3, 6} shown separately in Supplementary Fig. S8b. Nevertheless, practically, this tessellation can be transformed into the triangular grid by adjusting the phase of one of the gratings. This can be understood by comparing Supplementary Fig. S8b with Supplementary Fig. S4b.

References

1. I. Amidror, *The Theory of the Moiré Phenomenon, Vol. I: Periodic Layers*, 2^nd^ ed. (Springer-Verlag, London, 2009).

2. V. Saveljev, S.-K. Kim and J. Kim, Moiré effect in displays: a tutorial, Optical Engineering **57**, 030803 (2018).

3. M.C. Hutley, R. Hunt, R.F. Stevens and P. Savandert, Moiré magnifier, Pure and Applied Optics **3**, 133-142 (1994).

4. H. Kamal, R. Volkel, and J. Alda, Properties of moiré magnifiers, Optical Engineering **37**, 3007-3014 (1998).

5. K. Creath and J.C. Wyant, Moiré and fringe projection techniques, ch. 16 in *Optical Shop Testing*, 2^nd^ Ed., pp. 653-685 (John Wiley & Sons, New York, 1992).

6. C.A. Walker (Ed.), *Handbook of Moiré Measurement* (IOP Publishing, Bristol and Philadelphia, 2004).

7. E. Gabrielyan, The basics of line moiré patterns and optical speedup, Preprint, at https://arxiv.org/ftp/physics/papers/0703/0703098.pdf (2007).

8. C. Li, Z. Liu, H. Xie, and D. Wu, Statistics-based electron Moiré technique: a novel method applied to the characterization of mesoporous structures, Nanoscale **6**, 13409-13415 (2014).

9. S. Rasouli1 and M.T. Tavassoly, Analysis of the moiré pattern of moving periodic structures using reciprocal vector approach, Journal of Physics: Conference Series **350**, 012032 (2012).

10. C.A. Sciammarella, Gap Effect on Moiré Patterns, Zeitschrift für angewandte Mathematik und Physik (ZAMP) **19**, 326–333 (1968).

11. V. Saveljev and S.-K. Kim, Simulation and measurement of moiré patterns at finite distance, Optics Express **20**, 2163-2177 (2012).

12. H.S.M. Coxeter, *Regular Polytopes*, (Methuen & Co., London, 1948).
